# Blood loss in one-stage bilateral total knee arthroplasty: cruciate-retaining vs. posterior stabilized. A propensity score-matched analysis

**DOI:** 10.1051/sicotj/2024056

**Published:** 2024-12-23

**Authors:** Artit Laoruengthana, Thanawat Tantimethanon, Nopparat Santisathaporn, Thisayapong Inta-ngam, Krit Pongpirul, Piti Rattanaprichavej

**Affiliations:** 1 Department of Orthopaedics, Faculty of Medicine, Naresuan University 99 Moo 9, Phitsanulok-Nakohn Sawan Road Mueang District Phitsanulok Thailand; 2 Department of Orthopedics, Kamphaeng Phet Hospital 428 Ratcha Damnoen 1 Road Mueang District Kamphaeng Province 62000 Thailand; 3 Center of Excellence in Preventive & Integrative Medicine, Faculty of Medicine, Chulalongkorn University 1873 Rama IV Rd Pathum Wan District Bangkok 10330 Thailand; 4 Department of Preventive and Social Medicine, Faculty of Medicine, Chulalongkorn University 1873 Rama IV Rd Pathum Wan District Bangkok 10330 Thailand; 5 Department of International Health, Johns Hopkins Bloomberg School of Public Health 621 N. Washington Street Baltimore MD 21205 USA

**Keywords:** Knee arthroplasty, Blood loss, Blood transfusion, Pain, Prostheses

## Abstract

*Introduction*: Although single-stage bilateral total knee arthroplasty (BTKA) presents several advantages, higher perioperative blood loss is a potentiate drawback that is still inevitable. Cruciate retaining (CR) TKA may theoretically result in less blood loss, offer better proprioception, and more physiologic kinematics compared to posterior stabilized (PS) TKA. The objective of this study was to compare perioperative blood loss and recovery among patients who underwent CR and PS BTKA. *Methods*: A cohort of 46 CR BTKA and 80 PS BTKA performed by a single surgeon were retrospectively evaluated. Identical surgical techniques and perioperative care were provided to all patients. Propensity score matching was utilized to compare blood loss, a visual analog scale (VAS) for postoperative pain level, morphine consumption, knee flexion arc, and length of stay (LOS). *Results*: Comparing CR BTKA and PS BTKA, drain output was 206.44 mL vs. 194.89 mL (*p* = 0.47), calculated blood loss was 886.23 mL vs. 724.89 mL (*p* = 0.05), and blood transfusion rate was 18% vs. 17% (*p* = 1.00). Additionally, CR BTKA had higher VAS than PS BTKA, at 6 h: 5.74 vs. 3.78 (*p* < 0.001), and at 12 h: 5.80 vs. 4.74 (*p* = 0.02). CR BTKA group had higher morphine consumption (26.87 mg vs. 19.74 mg; *p* = 0.01) in the first 48 h. CR BTKA showed significantly less knee flexion angle during 48–72 h postoperative. *Conclusions*: The use of the CR prosthesis in BTKA could not demonstrate a superiority over the PS design in terms of blood loss, and recovery of knee function during the acute postoperative period.

## Introduction

Approximately 20% of patients with osteoarthritic knee present with bilateral involvement is severely enough to necessitate a single-stage bilateral total knee arthroplasty (BTKA) [1]. Currently, BTKA has been increasingly performed by knee arthroplasty surgeons because it has been known to offer several advantages such as single anesthesia and hospitalization, reduced overall recovery time, decreased malfunction during a staged procedure, and decreased overall cost while maintaining comparable functional outcomes [2].

However, some drawbacks, including greater perioperative blood loss and increased postoperative blood transfusion requirements, are still inevitable in BTKA settings. Recent published articles found that blood loss after BTKA ranged between 874 and 1,067 mL, and the transfusion rate is ranging between 40% and 53% [3–5]. This substantial blood loss after BTKA may cause hypoperfusion of vital organs, and blood transfusions may increase the risk of cardiopulmonary complications [6]. Additionally, the blood loss after total knee arthroplasty (TKA) can be concealed in a third space and may aggravate the inflammatory process as well as post-TKA pain [7, 8].

For TKA, two main systems that are commonly employed by surgeons are the cruciate retaining (CR) TKA and the posterior stabilized (PS) TKA. The choice between these systems is often selected based on individual preferences and the stability of patient’s knee. Some potential benefits including improved proprioception, more physiologic kinematics, and more bone preservation have been proposed for CR TKA [9–11]. CR TKA may theoretically result in less blood loss due to reduced bone excision. However, the actual impact on blood loss remains uncertain as studies have yielded equivocal findings [12, 13]. The comparison between CR and PS TKA also remains inconclusive regarding their differences in postoperative pain and recovery in the early postoperative phase [9, 14–16]. Particularly in the BTKA setting, there is limited evidence determining the superiority of either CR or PS design. Hence, the objective of the present investigation was to determine the difference of perioperative blood loss between CR and PS BTKA as a primary outcome, and secondary outcomes were the distinction of postoperative pain and functional recovery. The author hypothesizes that performing CR instead of PS BTKA may help reduce blood loss and enhance early recovery.

## Materials and methods

### Inclusion criteria

This study was a retrospective study of patients who received BTKA with either PS BTKA or CR BTKA by a single surgeon. The surgeon shifted from PS BTKA to perform consecutive CR BTKA during 2021–2023. Every patient with a diagnosis of end-stage bilateral primary osteoarthritis of knees who agree to undergone bilateral TKA in a single anesthesia were enrolled. Patients with prior knee infection, previous knee surgery history, and severe hip pathology that limit range of motion were excluded. Patients with insufficient data and preoperative hemoglobin less than 10 g/dL were also excluded. Forty-six consecutive patients who underwent CR BTKA during 2021–2023 and a cohort of 80 consecutive patients who received PS BTKA during 2018–2021 were compared. This study was approved by the Institutional Review Board, and informed consent was elicited from every patient.

### Surgical techniques

Neuraxial anesthesia consisting of Bupivacaine (0.5% Marcaine, AstraZeneca, Sweden), prophylactic intravenous antibiotic, and tourniquet settled at 250 mmHg, were utilized to all patients. All surgical procedures were performed by a single surgeon with an identical surgical technique through a standard medial parapatellar approach with approximately 10 cm of skin incision. Following an arthrotomy, a distal femoral cut was performed with an intramedullary device, whereas an extramedullary reference system was applied for proximal tibial cut. The soft tissue was released and balanced to achieve appropriate flexion and extension gap. The patella would be resurfaced when there is notably damaged cartilage with bony eburnation or the present of patellar maltracking, however, all the patella was not resurfaced in the present study. After finishing all the bone cuts, the entry point of the femoral medullary canal was occluded with a piece of autologous bone. A periarticular injection (PAI) mixture containing 50 mg of Bupivacaine (0.5% Marcaine; AstraZeneca, Sweden), and sterile normal saline solution, was then injected into the operated knees. Patients in the PS BTKA group were implanted with cemented, fixed-bearing, posterior stabilized prostheses (63 BTKA with NexGen LPS, Zimmer Biomet, Warsaw, IN, USA, and 17 BTKA with Vanguard, Zimmer Biomet, Warsaw, IN, USA), while patients in the CR BTKA group had cemented, fixed-bearing, cruciate-retaining prostheses (26 BTKA with (PFC Sigma, DePuy Synthes, Warsaw, IN, USA, and 20 BTKA with Vanguard, Zimmer Biomet, Warsaw, IN, USA). Before the arthrotomy closure, the negative pressure drain was placed into the joint, and topical tranexamic acid was applied into the joint. A compressive dressing was applied while the drain was clamped for 3 hours (h) and kept for 24 h.

Identical postoperative protocol was conducted for every patient. For the first 48 h, an intravenous patient-controlled analgesia (PCA) morphine (100 mL solution containing 50 mg of morphine sulphate) was offered as an on-demand bolus of 1 mL with a 5-minute lockout period, ketorolac (30 mg) was administered intravenously every 8 h, and oral acetaminophen (500 mg) was given thrice a day. Thereafter, 2 mg of morphine were additionally used for breakthrough pain every 4 h. The serum Hb level was monitored at 24, 48, and 72 h after the index procedure. Rehabilitation was started on the next day to promote knee movement and early ambulation. All patients received low molecular weight heparin for the first 48 h, and patient at risk for VTE would continue oral warfarin for 2 weeks.

### Outcomes measurement

The patient’s total blood volume (TBV) was calculated using the equation of Nadler et al. [17]. The difference between preoperative and lowest postoperative hemoglobin (Hb) was applied using the hemoglobin balance method to determine calculated blood loss (CBL) [18] (Table 1). A serum Hb level less than 9.0 g/dL is triggered for a blood transfusion at our institution. All the outcome were evaluated by assessors who were blinded to the treatment protocol.

**Table 1:** Total blood volume (TBV) and Hemoglobin balance method to determine calculated blood loss (CBL).


Male: TBV (mL) = (0.0003669 × height^3^ [cm]) + (32.19 × body weight [kg]) + 604
Female: TBV (mL) = (0.0003561 × height^3^ [cm]) + (33.08 × body weight [kg]) + 183
Calculated blood loss (mL) = TBV [mL] × (Hb_i_ – Hb_e_)/Hb_i_ + sum of blood products transfused [mL]
Hb_i_ [g/dL] was defined as the preoperative Hb, and Hb_e_ [g/dL] was the postoperative Hb.

The primary outcomes of this study are a comparison of drain output, CBL, and allogeneic blood transfusion rates between the PS BTKA and CR BTKA groups. Secondary outcomes including of operative duration, drainage output, the pain intensity determined by the 10-cm visual analog scale (VAS), morphine consumption, ability to achieve straight leg raise (SLR), maximal angle of knee flexion, length of stay (LOS), preoperative and 1 year postoperative Oxford Knee Score (OKS) which were prospectively collected at our hospital, were also compared between groups.

## Statistical analysis

Data were assessed by using Stata version 17.0 (StataCorp., College Station, TX, USA). Statistical significance was defined as a two-tailed *p-*value less than 0.05. Descriptive statistics were used to describe the prognostic factors. Categorical data are expressed as frequencies and percentages, while continuous data are presented as means and standard deviations. The standardized difference (STD) was applied to evaluate differences in each variable between the groups. An absolute STD value of less than 10% indicated no significant difference between the groups. To reduce the biases, propensity scores were employed to match the two groups based on prosthesis design. Propensity scores were derived from multiple logistic regression and the model incorporated sex, age, body mass index (BMI), American Society of Anesthesiologists (ASA) physical status classification, and preoperative Hb, and then propensity scores were divided into ten blocks. The matching method was the nearest neighbor algorithm, 1:1 ratio, within each block. Thereafter, the balance of baseline characteristics between the two groups was assessed using STD, to ensure the minimization of the bias. Finally, multivariable Gaussian regression was adjusted to assess mean calculated blood loss, VAS score, Knee flexion, morphine consumption, LOS, date of independent ambulation, operative time and drain in 24 h. Multivariable linear regression was applied to compare the risk of transfusion and SLR between the groups.

After the propensity matching, the cohort of PS BTKA and CR BTKA groups had > 80% power to detect a difference of 200 mL in CBL with a standard deviation (SD) of 300 mL, and > 80% power for 1.5 difference of VAS with SD of 2.0, respectively, with type I error of 5%.

## Results

After propensity score matching, 43 patients of each group were enrolled for final assessment. The demographic data including age, gender, BMI and ASA scores of these two groups were comparable after matching. Moreover, no significant differences between groups were identified in terms of preoperative VAS, ROM and Hb level (Table 2).

**Table 2:** Demographic data and preoperative characteristics of the PS and CR BTKA groups before and after propensity score matching.

Variables	Before propensity score match			After propensity score match		
	PS BTKA (*n* = 80)	CR BTKA (*n* = 46)	*p* value	PS BTKA (*n* = 43)	CR BTKA (*n* = 43)	*p* value
Age						
– Mean ± SD	65.74 ± 6.47	65.87 ± 8.94	0.92	66.44 ± 6.80	65.84 ± 8.94	0.72
Gender						
Male (%)	8 (10)	9 (19.57)		6 (13.95)	6 (13.95)	
Female (%)	72 (90)	37 (80.43)	0.18	37 (86.05)	37 (86.05)	1.0
BMI (kg/m^2^)						
– Mean ± SD	26.53 ± 3.96	26.83 ± 4.09	0.69	26.44 ± 3.84	26.68 ± 4.09	0.78
ASA scores						
1 (%)	0	0		0	0	
2 (%)	44 (55.00)	21 (45.65)		19 (44.19)	20 (46.51)	
3 (%)	36 (45.00)	25 (54.35)		24 (55.81)	23 (53.49)	
Preoperative VAS	7.33 ± 1.57	7.18 ± 1.46	0.62	7.40 ± 1.41	7.01 ± 1.34	0.20
Preoperative ROM (°)	108.23 ± 15.90	109.22 ± 12.82	0.70	107.00 ± 13.25	107.58 ± 15.89	0.85
Preoperative hemoglobin (g/dL)	12.50 ± 1.40	12.41 ± 1.17	0.68	12.17 ± 1.33	12.47 ± 1.40	0.30
Preoperative OKS	12.60 ± 4.99	13.0 ± 4.31	0.75	12.85 ± 4.64	13.30 ± 4.70	0.76

Data are presented with mean ± SD, except Gender, and American Society of Anesthesiologists scores (ASA). *n* = number, BMI = body mass index, kg = kilogram, m = meter, kg/m^2^ = kilogram/meter^2^, VAS = visual analog scales for pain intensity, ROM = range of knee motion, ° = degree, g/dL = gram/deciliter, OKS = Oxford Knee Score. Statistically significant (*p* < 0.05).

### Primary outcomes

Comparing CR BTKA and PS BTKA, drain output was 206.44 mL vs. 194.89 mL (*p* = 0.47), CBL was 886.23 mL vs. 724.89 mL (*p* = 0.05), and blood transfusion rate was 18% vs. 17% (*p* = 1.00) (Table 3). After multivariable regression, the CR BTKA demonstrated a comparable allogenic blood transfusion rate to PS BTKA (risk ratio = 1.12; 95% CI 0.70–1.81, *p* = 0.63).

**Table 3:** Peri- and post-operative outcomes of the PS and CR BTKA groups after propensity score matching.

	PS BTKA (*n* = 43) mean (95%CI)	CR BTKA (*n* = 43) mean (95%CI)	*p* value
Operative time (min)	124.24 (119.23–129.24)	117.83 (112.83–122.83)	0.08
Drain output (mL)	194.89 (172.62–217.17)	206.44 (184.17–228.72)	0.47
Calculated blood loss (CBL) (mL)	724.89 (620.19–829.58)	886.23 (765.55–1006.91)	0.05
Blood transfusion (%)	17 (39.53)	18 (41.86)	1.0
Visual analog scale for postoperative pain intensity			
6 h	3.78 (3.16–4.40)	5.74 (5.12–6.36)	<0.01*
12 h	4.74 (4.11–5.36)	5.80 (5.17–6.43)	0.02*
24 h	4.47 (3.83–5.11)	4.26 (3.63–4.90)	0.66
48 h	3.88 (3.22–4.53)	4.51 (3.86–5.16)	0.18
72 h	3.28 (2.61–3.95)	3.95 (3.28–4.62)	0.17
96 h	2.63 (1.94–3.32)	3.02 (2.33–3.71)	0.44
Morphine consumption (mg)			
24 h	14.86 (11.82–17.89)	19.67 (16.64–22.71)	0.03*
48 h	19.74 (15.82–23.67)	26.87 (22.95–30.80)	0.01*
Knee flexion angle (°)			
24 h	55.92 (51.03–60.80)	59.33 (54.44–64.21)	0.34
48 h	75.44 (70.56–80.33)	66.60 (61.72–71.49)	0.01*
72 h	86.79 (81.91–91.68)	79.53 (74.65–84.42)	0.04*
96 h	90.41 (85.53–95.30)	86.31 (81.43–91.20)	0.25
LOS (days)	5.70 (5.23–6.17)	5.58 (5.11–6.04)	0.70
OKS at 1 year of follow-up	42.45 ± 2.21	42.3 ± 2.32	0.84

min = minutes, mL = milliliters, h = hours, mg = milligrams, ° = degree, PONV = postoperative nausea vomiting, LOS = length of stay, 95% CI = 95% confidence interval. * Statistically significant (*p* < 0.05), OKS = Oxford Knee Score.

### Secondary outcomes

**Figure 1:**
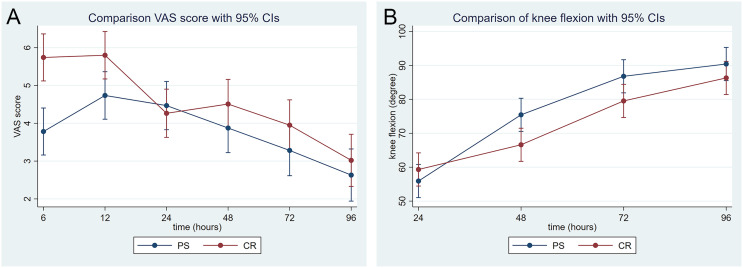
Postoperative pain intensity and knee flexion arc. (a) Visual analog scales (VAS) for pain intensity assessed at 6 h, 12 h, 24 h, 48 h, 72 h, and 96 h postoperative. (b) Knee flexion angle assessed at 24 h, 48 h, 72 h, and 96 h postoperative. PS = PS BTKA group, CR = CR BTKA group, 95 % CI = 95 % confidence interval.

CR BTKA had higher VAS of postoperative pain intensity than PS BTKA, particularly during the first 12 h (at 6 h: 5.74 vs. 3.78; *p* < 0.01, at 12 h: 5.80 vs. 4.74; *p* = 0.02). Nevertheless, this significant difference in postoperative pain intensity was not detected after 12 h postoperative (Figure 1A). Additionally, the CR TKA group had higher morphine consumption than the PS BTKA group at either 24 h (19.67 mg vs. 14.80 mg; *p* = 0.03) or 48 h (26.87 mg vs. 19.74 mg; *p* = 0.01) after the surgery (Table 3).

In terms of knee flexion, PS BTKA proposed a trend of better flexion arc, especially at 48 h postop (75.44° vs. 66.60°; *p* = 0.01) and 72 h postop (86.79° vs. 79.53°; *p* = 0.04) (Figure 1B). The risk ratio of ability to achieve SLR of CR BTKA was 0.89 (95% CI 0.60–1.34, *p* = 0.58) at 24 h, 0.86 (95% CI 0.59–1.24, *p* = 0.42) at 48 h, 0.84 (95% CI 0.60–1.16, *p* = 0.42) at 72 h, and 0.89 (95% CI 0.71–1.13, *p* = 0.34) at 96 h postoperative. Other secondary outcomes such as operative time, OKS, and LOS were comparable among these two groups (Table 3).

## Discussion

In the present study, CR BTKA demonstrated comparable primary outcomes to PS BTKA including drain output, CBL and transfusion rate. Nonetheless, CR BTKA showed a significantly higher VAS pain score at 6, 12 h postoperatively with increased morphine consumption at 24 and 48 h after the surgery compared to PS BTKA. Additionally, CR BTKA proposed a significantly lesser knee flexion angle at 48 and 72 h postoperatively, however OKS was not different between groups at 1 year of follow up.

Theoretically, performing CR TKA may reduce blood loss and enhance recovery during the acute postoperative period [9, 12, 14]. Some previous studies have compared the amount of blood loss and transfusion rate between CR and PS unilateral TKA [12, 13]. Retrospective study of 233 PS TKA and 240 CR TKA that were from the same manufacturer revealed that the PS TKA group significantly had a greater amount of blood loss than the CR TKA group, even if there was no difference observed in terms of blood transfusion [12]. Whereas, Cankaya et al. [13] found that PS unilateral TKA had approximately 70 mL higher CBL than CR unilateral TKA, but this difference did not reach a statistical significance. Thus, while controversy remains over benefit of CR prosthesis for unilateral TKA in terms of blood loss, evidence comparing blood loss between CR and PS in BTKA setting is limited. Our findings suggest that less bone excision of CR prosthesis could neither reduce perioperative blood loss nor blood transfusion for BTKA. On the other hand, other applications such as bone wax, platelet-rich plasma, and closed-box knee prosthesis which has been used as sealant for bleeding bone or a covering for exposed bone may be alternative strategies to reduce ongoing blood loss after TKA [4, 19, 20].

For secondary outcomes, pain intensity and recovery of knee function in the acute postoperative phase between CR and PS TKA are seldom reported, especially in BTKA. Fiedler et al. [21] recently published a retrospective propensity score match comparing between 616 CR TKA to 616 PS TKA. Their results showed no significant difference among CR TKA and PS TKA in terms of immediate postoperative pain and morphine consumption during 72 h postoperatively. For BTKA, we found the outcomes of CR prosthesis in terms of immediate postoperative pain, total morphine use, and knee flexion arc were inferior to PS implant. Thus, we hypothesized that the preservation of PCL may conversely become the pain generator because of the increase of PCL tension during femoral rollback, following CR TKA.

CR prosthesis has been considered to offer better proprioception, femoral rollback, and quadriceps recovery than PS TKA [9–11, 22]. Andriacchi and Galante [22] noted the femoral rollback caused by the PCL can enhance the quadriceps mechanism during knee flexion as the lever arm changes up to 40%. Warren et al. [10] reported increased joint position awareness in CR TKA because of the retention of mechanoreceptors within the PCL. However, recent literature indicates no significant differences in quadriceps strength, and functional scores between the CR and PS designs during 6 months to 2 years of follow-up [16, 23], which is accord to our OKS finding. Conversely, some prospective randomized study and meta-analyses suggest that PS TKA may show greater improvement in functional scores and a larger flexion arc compared to CR TKA [14, 15, 24, 25].

There are some limitations in this study. The first limitation is associated to the retrospective study design which may introduce inherent biases. Nevertheless, to address potential confounding factors between patient groups, a propensity score-matched analysis was employed to deduct those factors between groups. As well, this study was conducted by a single surgeon which may enhance internal validity. Second, this study is vulnerable to selection bias due to the use of each prosthesis design in different timeframes and may be related to unequal representation of certain patient groups. Third, although no patients in this study received patellar resurfacing, Akti et al. [26] revealed that an addition of patellar resurfacing does not affect drainage volume, hidden blood loss, total blood loss and amount of blood transfusion compared to patellar non-resurfacing.

## Conclusion

The use of the CR prosthesis in BTKA could not demonstrate a superiority over the PS design in terms of blood loss and blood transfusion rate. Additionally, CR BTKA seems to have significantly higher postoperative pain scores and morphine consumption, and less knee flexion angle during the acute postoperative phase than experienced with PS BTKA.

## Data Availability

The data that support the findings of this study are available from the corresponding author upon reasonable request.
